# Supplementary material for: Measuring the frequency and determinants of COVID-19 prevention behaviours: a cross-sectional assessment of large-scale programmes in seven countries, late 2020
Source: BMJ Open. 2024 Aug 16;14(8):e082419. doi: 10.1136/bmjopen-2023-082419 (PMC11331854; doi:10.1136/bmjopen-2023-082419)
Supplement: Supplementary file 4 [file bmjopen-2023-082419.reviewer_comments.pdf]

PEER REVIEW HISTORY

BMJ Open publishes all reviews undertaken for accepted manuscripts. Reviewers are asked to complete a checklist review form (**http://bmjopen.bmj.com/site/about/resources/checklist.pdf**) and are provided with free text boxes to elaborate on their assessment. These free text comments are reproduced below.

ARTICLE DETAILS

**TITLE (PROVISIONAL)**	Measuring the frequency and determinants of COVID-19 prevention behaviours: a cross-sectional assessment of large-scale programmes in seven countries, late 2020
**AUTHORS**	Bick, Sarah; White, Sian; Thorseth, Astrid; Friedrich, Max; Gavin, Ian; Prasad-Gautam, Om; Dreibelbis , Robert

VERSION 1 – REVIEW

**REVIEWER**	Oyenubi, Adeola<br>University of the Witwatersrand Johannesburg, School of Economics and Finance
**REVIEW RETURNED**	07-Jan-2024

**GENERAL COMMENTS**	This paper examines the vulnerability of residents of LMIC in respect of access to hygiene facilities, water, soap and masks, and dense living environments and how these factors impede effective practise of preventive behaviour in the context of COVID-19.<br>The paper analyses a multi-country data (Ethiopia, Ghana, Nepal, Nigeria, Rwanda, Tanzania and Zambia). I think the papers cover important issues that relates to the topic in developing contexts.<br><br>The strength of the paper includes its focus on behavioural aspects of preventive measures (knowledge, norms, barriers, motives), the use of multi-country data and the use of face-to-face surveys. I think the exploration of handwashing moments is particularly important because it speaks to behavoural factors (the show that despite general increase in handwashing this tend not to occur at important moments that decrease the spread of the virus).<br>The only thing I think is missing is a proper explanation of heterogeneity in preventive bahaviour across countries. What are the contextual factors that this heterogeneity may be attributed to. For example, it has been found that cultural context matters when it comes to the relationship between health communication and compliance Ying Li et al (2022). I know there was a discussion about stringency index (which I assume this is from the "our world in data" database) a variable that was not introduced until the discussion part of the paper (which makes things a little bit confusing) but that explanation is coming latter than it should in my opinion. Further even with variation in stringency index compliance might depend on contexts across countries.<br>While I don't see this as a major problem, introducing this variable earlier in the draft and discussing how it explains compliance

	behaviour is relevant to policy in terms of how to react to similar health emergency should one occur.<br>A related point is how these countries compare? for example including a country dummy in the regression analysis may shed some light on this.<br><br><br>Ying Li, Luan, S., Li, Y., Wu, J., Li, W., & Hertwig, R. (2022). Does risk perception motivate preventive behavior during a pandemic? A longitudinal study in the United States and China. American Psychologist, 77(1), 111

**REVIEWER**	Lawson, Aaron<br>Ulster University
**REVIEW RETURNED**	08-Mar-2024

**GENERAL COMMENTS**	Title: Frequency and determinants of COVID-19 prevention behaviours: assessment of large-scale programmes in seven countries<br>This study describes a multi-country analysis of prevalence of key hygiene prevention behaviours and their determinants associated with an international non-governmental organisation (WaterAid) hygiene behaviour change programmes for COVID-19 prevention. The goal of this analysis is to inform future outbreak preparedness and pandemic response in LMICs.<br>The study has relevance to public health, communicable disease transmission and the importance of personal public health and sanitation behaviours to mitigate against COVID-19.<br>The study methodology relied on a quantitative approach utilising cross-sectional household surveys in seven countries where WaterAid worked (Ethiopia, Ghana, Nepal, Nigeria, Rwanda, Tanzania and Zambia).<br>The presentation of the results was good and there was a clear link between the methodology, results and discussion of findings. Overall the paper presented is well written, and there are some useful findings in relation to behavioural considerations and in preparing for future pandemics.

VERSION 1 – AUTHOR RESPONSE

Reviewer 1 comments:

This paper examines the vulnerability of residents of LMIC in respect of access to hygiene facilities, water, soap and masks, and dense living environments and how these factors impede effective practise of preventive behaviour in the context of COVID-19.
The paper analyses a multi-country data (Ethiopia, Ghana, Nepal, Nigeria, Rwanda, Tanzania and Zambia). I think the papers cover important issues that relates to the topic in developing contexts.

The strength of the paper includes its focus on behavioural aspects of preventive measures (knowledge, norms, barriers, motives), the use of multi-country data and the use of face-to-face surveys. I think the exploration of handwashing moments is particularly important because it speaks

to behavoural factors (the show that despite general increase in handwashing this tend not to occur at important moments that decrease the spread of the virus).
The only thing I think is missing is a proper explanation of heterogeneity in preventive bahaviour across countries. What are the contextual factors that this heterogeneity may be attributed to. For example, it has been found that cultural context matters when it comes to the relationship between health communication and compliance Ying Li et al (2022). I know there was a discussion about stringency index (which I assume this is from the "our world in data" database) a variable that was not introduced until the discussion part of the paper (which makes things a little bit confusing) but that explanation is coming latter than it should in my opinion. Further even with variation in stringency index compliance might depend on contexts across countries.
While I don't see this as a major problem, introducing this variable earlier in the draft and discussing how it explains compliance behaviour is relevant to policy in terms of how to react to similar health emergency should one occur.

Response: Thank you for your thoughtful and relevant comments. The stringency index variable was indeed taken from the "our world in data" database, which we have referenced. Based on your feedback we have conducted additional analyses adjusting for stringency index at the mid-point of our data collection period to understand whether this variable explains some of the variation in outcomes. This variable was not significantly associated with any of our primary outcomes and adjusting for it did not alter the associations with any other determinants of self-reported behaviour. There was no information gained by including this variable and we have retained the outputs from the original analyses. We have added description to the methods (page 13), results (page 20) and discussion (page 21).

We recognise that this was only one indicator combining multiple national contextual factors taken at a single time-point (one datapoint per country), and there are many contextual factors that may moderate relationships between health communication and compliance. We have acknowledged this limitation on page 24 (lines 488–491) of the revised manuscript.

A related point is how these countries compare? for example including a country dummy in the regression analysis may shed some light on this.

Response: We used random effects models to account for between-country variation in outcomes instead of including country as a fixed effect. We present descriptive statistics on primary outcomes across countries on pages 14 and 15, but making formal comparisons between countries may have been misleading given diverse sampling methods and locations studied, and led to mistaken interpretations of adjusted effects of different countries in multivariable models (Table 2 fallacy).

Reviewer 2 comments:

This study describes a multi-country analysis of prevalence of key hygiene prevention behaviours and their determinants associated with an international non-governmental organisation (WaterAid) hygiene behaviour change programmes for COVID-19 prevention. The goal of this analysis is to inform future outbreak preparedness and pandemic response in LMICs.
The study has relevance to public health, communicable disease transmission and the importance of personal public health and sanitation behaviours to mitigate against COVID-19.

The study methodology relied on a quantitative approach utilising cross-sectional household surveys in seven countries where WaterAid worked (Ethiopia, Ghana, Nepal, Nigeria, Rwanda, Tanzania and Zambia).
The presentation of the results was good and there was a clear link between the methodology, results and discussion of findings. Overall the paper presented is well written, and there are some useful findings in relation to behavioural considerations and in preparing for future pandemics.

Response: We thank the reviewer for their encouraging feedback.

VERSION 2 – REVIEW

**REVIEWER**	Oyenubi, Adeola<br>University of the Witwatersrand Johannesburg, School of Economics and Finance
**REVIEW RETURNED**	08-Jul-2024

**GENERAL COMMENTS**	Thank you for addressing the comment.

VERSION 2 – AUTHOR RESPONSE
